# Chromosomal instability in the prediction of pituitary neuroendocrine tumors prognosis

**DOI:** 10.1186/s40478-020-01067-5

**Published:** 2020-11-10

**Authors:** Hélène Lasolle, Mad-Hélénie Elsensohn, Anne Wierinckx, Eudeline Alix, Clément Bonnefille, Alexandre Vasiljevic, Christine Cortet, Bénédicte Decoudier, Nathalie Sturm, Stephan Gaillard, Amandine Ferrière, Pascal Roy, Emmanuel Jouanneau, Philippe Bertolino, Claire Bardel, Damien Sanlaville, Gérald Raverot

**Affiliations:** 1 Fédération d’endocrinologie, Centre de Référence des Maladies Rares Hypophysaires, Groupement Hospitalier Est, Hospices Civils de Lyon, 8 av Doyen Lepine, 69677 Bron Cedex, France; 2 Université Lyon 1, Lyon, France; 3 INSERM U1052, CNRS UMR5286, Cancer Research Center of Lyon, 69372 Lyon, France; 4 Service de Biostatistique-Bioinformatique, Hospices Civils de Lyon, Lyon, France; 5 Équipe Biostatistique-Santé, Laboratoire de Biométrie et Biologie Évolutive, CNRS UMR 5558, Villeurbanne, France; 6 ProfileXpert, SFR-Est, CNRS UMR-S3453, INSERM US7, Lyon, France; 7 Service de Cytogénétique, Centre de Biologie et Pathologie Est, Hospices Civils de Lyon, Bron, France; 8 Centre de Pathologie Est, Groupement Hospitalier Est, Hospices Civils de Lyon, Bron, France; 9 Service d’Endocrinologie, CHRU de Lille, Hopital Huriez, 59037 Lille Cedex, France; 10 Service d’Endocrinologie - Diabète – Nutrition, Centre Hospitalier Universitaire de Reims, Reims, Champagne-Ardenne, France; 11 Département d’Anatomie et cytologie pathologique, CHU Grenoble Alpes, Grenoble, France; 12 Department of Neurosurgery, Foch Hospital, 92151 Suresnes, France; 13 Department of Endocrinology, Diabetes and Nutrition, CHU de Bordeaux, Bordeaux, France; 14 University of Bordeaux, 146 R Leo saignat, 33000 Bordeaux, France; 15 Service de Neurochirurgie, Groupement Hospitalier Est, Hospices Civils de Lyon, Bron, France; 16 Plateforme de séquençage haut débit, Hospices Civils de Lyon, Bron, France

**Keywords:** Pituitary neuroendocrine tumors, Prognosis, Genomic instability, Copy number variations, Pituitary adenoma

## Abstract

The purpose of this study was to analyze the impact of copy number variations (CNV) on sporadic pituitary neuroendocrine tumors (PitNETs) prognosis, to identify specific prognosis markers according to the known clinico-pathological classification. CGH array analysis was performed on 195 fresh-frozen PitNETs (56 gonadotroph, 11 immunonegative, 56 somatotroph, 39 lactotroph and 33 corticotroph), with 5 years post-surgery follow-up (124 recurrences), classified according to the five-tiered grading classification (invasion, Ki-67, mitotic index and p53 positivity). Effect of alterations on recurrence was studied using logistic regression models. Transcriptomic analysis of 32 lactotroph tumors was performed. The quantity of CNV was dependent on tumor type: higher in lactotroph (median(min–max) = 38% (0–97) of probes) compared to corticotroph (11% (0–77)), somatotroph (5% (0–99)), gonadotroph (0% (0–10)) and immunonegative tumors (0% (0–17). It was not predictive of recurrence in the whole cohort. In lactotroph tumors, genome instability, especially quantity of gains, significantly predicted recurrence independently of invasion and proliferation (p-value = 0.02, OR = 1.2). However, no specific CNV was found as a prognostic marker. Transcriptomic analysis of the genes included in the CNV and associated with prognosis didn’t show significantly overrepresented pathway. In somatotroph and corticotroph tumors, USP8 and GNAS mutations were not associated with genome disruption or recurrence respectively. To conclude, CGH array analysis showed genome instability was dependent on PitNET type. Lactotroph tumors were highly altered and the quantity of altered genome was associated with poorer prognosis though the mechanism is unclear, whereas gonadotroph and immunonegative tumors showed the same ‘quiet’ profile, leaving the mechanism underlying tumorigenesis open to question.

**Electronic supplementary material:**

The online version of this article (10.1186/s40478-020-01067-5) contains supplementary material, which is available to authorized users.

## Introduction

Pituitary neuroendocrine tumors (PitNETs) represent 10–15% of intra-cranial tumors among which most are benign and controlled by current therapeutic strategies. While surgery is the first-line treatment, it can also be associated with medical therapies. Despite these strategies, approximately 25–40% of PitNETs present a regrowth after surgery [15]. PitNETs that are recurrent and resistant to conventional treatment are considered as aggressive, however there is no standardized criteria to define them [21]. Various approaches have been proposed for the prediction of tumor behavior, including the study of pathological markers. While the recent WHO 2017 classification of PitNETs did not propose individual markers, it did identify a group of tumors with a high risk of recurrence, including sparsely granulated somatotroph tumors, lactotroph tumors in men, Crooke’s cell tumors, silent corticotroph tumors (SCT), and the newly introduced pluri-hormonal Pit-1-positive tumor [10]. Although interesting, the clinical impact of these groups of tumors is still limited since they represent a small number of cases and are not representative of the outcomes of the most common types of PitNETs.

Combining radiological and pathological characteristics, we have proposed a clinico-pathological prognosis classification, based on five grades, that associate proliferative (mitosis, Ki67, p53 expression) and invasiveness (cavernous or sphenoid sinus) criteria [32]. According to this classification grade 2b (invasive and proliferative tumors) presented higher risk of recurrence or progression on medical treatment compared to non-invasive non-proliferative tumors (grade 1a). The prognostic value of this classification has been validated by independent prospective study [22] and retrospective studies [1, 14] in all PitNETs types.

In parallel to studies on pathological markers, numerous studies have been conducted to identify genomic alterations leading to pituitary tumorigenesis and/or associated with PitNETs behavior [27]. Familial forms of PitNETs due to germinal mutations are more prone to resistance to medical treatment, however, the incidence of aggressive PitNETs or carcinomas did not appear to be higher compared to sporadic tumors [21]. Studies focusing on somatic mutations identified a low mutation rate [4, 16, 26]. *GNAS* gain-of-function mutations have been identified in 30% of somatotropinomas and *USP8 or USP48* mutations in about 40% of corticotroph PitNETs [6, 23, 25]. However, none of these mutations has been clearly associated with tumor behavior.

Since chromosome imbalance is frequent in tumors and associated with prognosis especially in brain tumors [2], several studies have analyzed the impact of such mechanisms on PitNETs. CGH analysis, performed using metaphase control chromosomes in a limited number of PitNETs, suggested that many alterations occur in PitNETs [19, 31]. Interestingly, those alterations may be preferentially found in functioning [28, 31] and in invasive PitNETs [28]. Whole exome sequencing analysis has pointed to the existence of 2 groups of PitNETs, defined as “disrupted” or “quiet”, depending on the quantity of copy number variations (CNV) [4]. These 2 groups were specifically associated with functional characteristics, and with GNAS mutation status in somatotroph tumors [3, 4, 9, 16, 26]. However, in most of these studies the non-functioning PitNETs were classified based on their clinical presentation and not on their histopathology. Such an approach led to a mix of gonadotroph, silent-corticotroph, -somatotroph, -lactotroph and non-immunoreactive tumors, as reported by Neou et al. [16].

Few studies have explored the direct association between CNV and recurrence. While a LOH analysis in non-functioning PitNETs found an increased frequency of 2 allelic losses on chromosome 1q in recurrent tumors [5], a CGH array performed on 13 lactotroph tumors showed recurrent loss of chromosome 11p in aggressive tumors compared to indolent tumors [36]. More recently, Neou et al. [16] reported the lack of association between aggressiveness and chromosomal alterations in 86 PitNETs of all types using whole exome sequencing. Unfortunately, this latter study was based on a cohort of patients with variable clinical data follow-up (1–120 months) and the definition of aggressiveness was not standardized. Finally, an association of chromosomal instability and markers of aggressiveness has also been reported in the subgroup of pediatric corticotroph PitNETs [30].

Since the genetic mechanisms underlying PitNETs growth and behavior are not fully understood, we conducted a large-scale CGHarray study to analyze the impact of CNV on sporadic PitNETs prognosis. Our aim was to identify specific markers associated with prognosis, in a large cohort of 195 PitNETs, taking into account the known clinico-pathological five-tiered classification and at least 5 years post-surgery follow-up.

## Methods

### Study design

The study is part of PITUIGENE, a French multicentric retrospective study (ClinicalTrials.gov Identifier: NCT01903967) based on 212 frozen surgical samples of PitNETs. Data are registered according the French data protection agency CNIL. Written informed consent was given by all patients, and the procedure was in accordance with the ethical standards and approved by a local ethics committee (committee for the protection of persons CPP SUD-EST IV LYON). Recruited patients presenting a lactotroph (PRL), somatotroph (GH), corticotroph (ACTH) and gonadotroph (FSH/LH) immunoreactive PitNET, were selected from 10 different centers in France. All patients were operated, via trans-sphenoidal route, between 1988 and 2010. Patients were selected based on a clinical follow-up of at least 5-years combined with the availability of matching frozen tumor samples. Patients who underwent adjuvant post-operative radiotherapy or presented germinal *MEN1* or *AIP* mutation were excluded. Forty-six patients were part of the HYPOPRONOS (PHRC 27–43) French multicenter retrospective study [22]. Tumors were classified as functional or silent PitNETs according to hormonal levels, i.e. plasma PRL for lactotroph tumors, IGF1 and GH levels for somatotroph tumors and urinary free cortisol and response to suppression tests for corticotroph tumors. For each patient, functional subtype and data on proliferation were recorded from histological evaluations as previously described [32]. Tumor with no hormone expression using immunohistochemistry analysis were classified as immunonegative.

### Definition of recurrence

Disease-free patients or patients controlled by medical treatment and/or with a stable remnant on MRI up to 5 years, were considered as non-recurring. Patients who presented recurrence/tumor progression on MRI and/or a significant increase of plasma hormone levels requiring therapeutic changes in the five years post-surgery were considered as recurring.

### CGH data

Tumor DNA was extracted from ~ 15 mg frozen tissue using MasterpureTM Complete DNA and RNA Purification Kit (Epicentre^®^ Biotechnologies, Madison, WI, USA). aCGH was performed using SurePrint G3 Human genome CGH + SNP Microarray, 4x180K (Agilent Technologies, Santa Clara, CA, USA). After enzymatic digestion by RsaI and AluI enzymes, 1.5 µg of tumor DNA and 0.6 µg of sex-matched human reference DNA (Agilent) were labelled by random priming with Cyanine 5 and Cyanine 3, respectively. Hybridization was performed at 65 °C for 24 h and the arrays scanned on Agilent DNA Microarray Scanner. Fluorescence was quantified with Feature Extraction 11.5.11 software whose output is the L2R (log2 (tumor DNA fluorescence)/(reference DNA fluorescence)) of each probe.

### *USP8* and *GNAS* sequencing

*GNAS* activating somatic mutations and *USP8* gain-of-function somatic mutations were determined using conventional Sanger DNA-sequencing in somatotroph and corticotroph tumors respectively. Genomic DNA was extracted from frozen tissue using MasterpureTM Complete DNA and RNA Purification Kit (Epicentre^®^ Biotechnologies, Madison, WI, USA). DNA sequences were amplified by PCR with the CORE 10 (Mpbio) NH4(SO4)2 Kit (MP Biomedicales) using forward 5′-CTATGTGCCGAGCGATCAGG-3′ and reverse 5′- CCGTGTGAATGCTTGGGAGA -3′ primers for *GNAS*, and forward 5′-CAACCTGAGATGCTGGCTAC-3′ and reverse 5′- CCAACTCCCTGACACTAACA-3′ primers for *USP8*. Sanger sequencing of PCR products was performed on a 3130xl Genetic Analyzer (Applied Biosystems^®^) following the use of BigDye™ Terminator v3.1 Cycle Sequencing Kit (Applied Biosystems^®^). Results were interpreted using Seqscape V3 software.

### Transcriptomic analysis

Transcriptomic analysis of 32 lactotroph PitNETs was done using CodeLink Uniset Human Whole Genome bioarrays containing 55,000 human oligonucleotide gene probes (GE Healthcare Europe GmbH, Freiburg, Germany), of which 16 were also analyzed by CGH array analysis. Technical details were previously described by Wierinckx et al. [35].

### Statistical analysis

#### CGH data preparation

For each patient, raw CGH data were normalized and subsequently centralized as reported previously [13]. The centralization step based on FISH analysis was applied on patient profiles with at least one alteration longer than 5 Mb. Circular binary segmentation was applied on the centralized and normalized Log2Ratios (L2R) (with significance level for the test to accept change-points = 10^−6^) [18]. X and Y chromosomes were excluded from the analysis. All identified CNV were manually reviewed by an experienced cytogenetician (EA). Loss of heterozygosity (LOH) calling was performed using Cytogenomics 3.0.3.3 software (Agilent) for tumors with derivative log ratio spread (DLRS) less than 0.3.

#### Descriptive analysis

Genome instability was determined using the number of altered (deleted + gained) probes compared to the total number of probes. The association between clinical data and quantity of altered probes was tested using Wilcoxon rank tests. Non-supervised hierarchical clustering was done using Jaccard distance and Ward criterion, by tumor type and in the whole cohort. The associations between clinical data and clusters, and between clinical data and *USP8* or *GNAS* mutations, were tested using Fisher and Kruskal–Wallis tests.

#### Analysis of prognosis

The association between tumor recurrence and the number of altered probes (deleted + gained, then deleted + gained + copy neutral LOH) was studied using univariate logistic regression models in the whole cohort, and then for each tumor type. Subsequently, multivariate analysis adjusted for the main known factors of recurrence (tumor type, histological grade, age at surgery and sex) were performed. In the per-type analysis, histological grades 1a and 1b were grouped due to the small number of 1b samples. For corticotroph and somatotroph tumors, an analysis adjusted to the presence of *USP8* and *GNAS* mutations respectively was also done. Likelihood ratio tests (LRT) were calculated to compare the models.

Specific alterations associated with recurrence were searched for through univariate and multivariate (including tumor type, histological grade, age at surgery and sex) logistic regressions for each probe (deleted, normal state or gained) in the whole cohort and in each tumor type separately. LRT were calculated to compare the models with and without the probe status. P-values were adjusted for dependent multiple testing using the Benjamini-Yekutieli approach.

In order to perform transcriptomic analysis comparisons in lactotroph tumors, genes included in CNV were listed using the Hg19 reference genome. For each gene (altered or non-altered), we performed univariate and multivariate (including clinical data) logistic regression models. LRT were calculated to compare models with and without the gene status. p-values were adjusted for multiple testing using the Benjamini–Hochberg approach.

#### Transcriptomic analysis

mRNA transcripts showing at least 2-fold variation were considered as differentially expressed after statistical analysis using Student’s t-test with a p-value ≤ 0.05. Gene Set enrichment analysis (GSEA) was performed to search for implicated biological pathways, following the recommended protocol from the Broad Institute Gene Set Enrichment Analysis website (https://www.gsea-msigdb.org/gsea). GSEA software v7.0.0 and the Molecular Signatures Database v7.0. were used for running GSEA. A ranked-list metric was generated by calculating the signal-to-noise ratio. The number of permutations was set to 1000. Nominal p-values < 0.05 and adjusted q-values (FDR) < 0.25 were considered as significant.

All analyses were performed with R software version 3.5.2. P-values and adjusted p-values less than 0.05 were considered as significant.

## Results

### Cohort description

Of the 212 PitNETs initially included, 17 tumors were excluded due to unsatisfactory sequencing quality (DLRS > 0.47) (n = 7); the presence of alterations larger than 5 MB and the lack of material to confirm centralization by FISH (n = 9), or missing information concerning their pathological grade (n = 1). A total of 195 PitNETs were analyzed, including 56 gonadotroph, 11 immunonegative, 56 somatotroph, 39 lactotroph and 33 corticotroph (8 being silent) tumors. Clinico-pathological characteristics of those tumors are presented in Table 1, their mean (sd) post-operative follow-up was 8.3(3.5) years and tumor recurrence/progression occurred in 124 patients (64%) within 1.4(1.6) years after initial surgery.

**Table 1:** Description of patients and tumors

	Recurrence	No recurrence	Analyzed cohort	Excluded
N	124	71	195	17
Age mean ± SD (years)	44.8 (14.4)	51.1 (13.4)	47.1 (14.3%)	43.3 (16.3)
Sex				
F	50 (40.3%)	34 (47.9%)	84 (43.1%)	8 (47.1%)
M	74 (59.7%)	37 (52.1%)	111 (56.9%)	9 (52.9%)
Tumor type				
Gonadotroph	33 (26.6%)	23 (32.4%)	56 (28.7%)	2 (11.7%)
Immunonegative	5 (4.0%)	6 (8.5%)	11 (5.6%)	1 (5.9%)
Somatotroph	40 (32.2%)	16 (22.5%)	56 (28.7%)	13 (76.5%)
GNASwt	31	9	40	/
GNAS mutation	7	6	13	/
GNAS Not available	2	1	3	/
Lactotroph	28 (22.6%)	11 (15.5%)	39 (20.0%)	0 (0%)
Corticotroph	18 (14.6%)	15 (21.1%)	33 (17.0%)	1 (5.9%)
USP8wt	12	10	22	/
USP8 mutation	3	2	5	/
USP8 Not available	3	3	6	/
Grade				
1a	26 (21.0%)	36 (50.7%)	62 (31.9%)	2 (13.3%)
1b	4 (3.2%)	5 (7.0%)	9 (4.6%)	1 (6.7%)
2a	64 (51.6%)	25 (35.3%)	89 (45.6%)	7 (46.7%)
2b	30 (24.2%)	5 (7.0%)	35 (17.9%)	5 (33.3%)
Size				
Microadenomas	4 (3.2%)	9 (12.7%)	13 (6.7%)	2 (11.8%)
Macroadenomas	111 (89.6%)	62 (87.3%)	173 (88.7%)	15 (88.2%)
Giant adenomas	8 (6.4%)	0	8 (4.1%)	0
Not available	1 (0.8%)	0	1 (0.5%)	0

Somatic pathogenic mutations of *USP8* were detected in 5/27 (19%) corticotroph tumors (heterozygous p.Ser718Pro (n = 2), p.Ser718Cys, p.Ser719del and p.Pro720Arg). In addition, two of the wild type *USP8* corticotroph tumors harbored a respective gain and deletion of 15q21.2 region. Somatic pathogenic mutations of *GNAS* were identified in 13/53 (25%) somatotroph tumors (11 heterozygous missense p.Arg201Cys mutations and 2 p.Gln227Leu). In addition, 13 *GNAS* wild-type somatotroph tumors showed a gain including the 20q13.32 region. These gains were confirmed using FISH analysis in 5/5 studied tumors (supplemental Figure 1). Tumors with *GNAS* mutation, tumors with gain of the *GNAS* region and tumors with no alteration (*GNAS* wt and no gain) were comparable in terms of sex, age at surgery, grade, secretion and tumor size.

### Genomic instability description

**Fig. 1:**
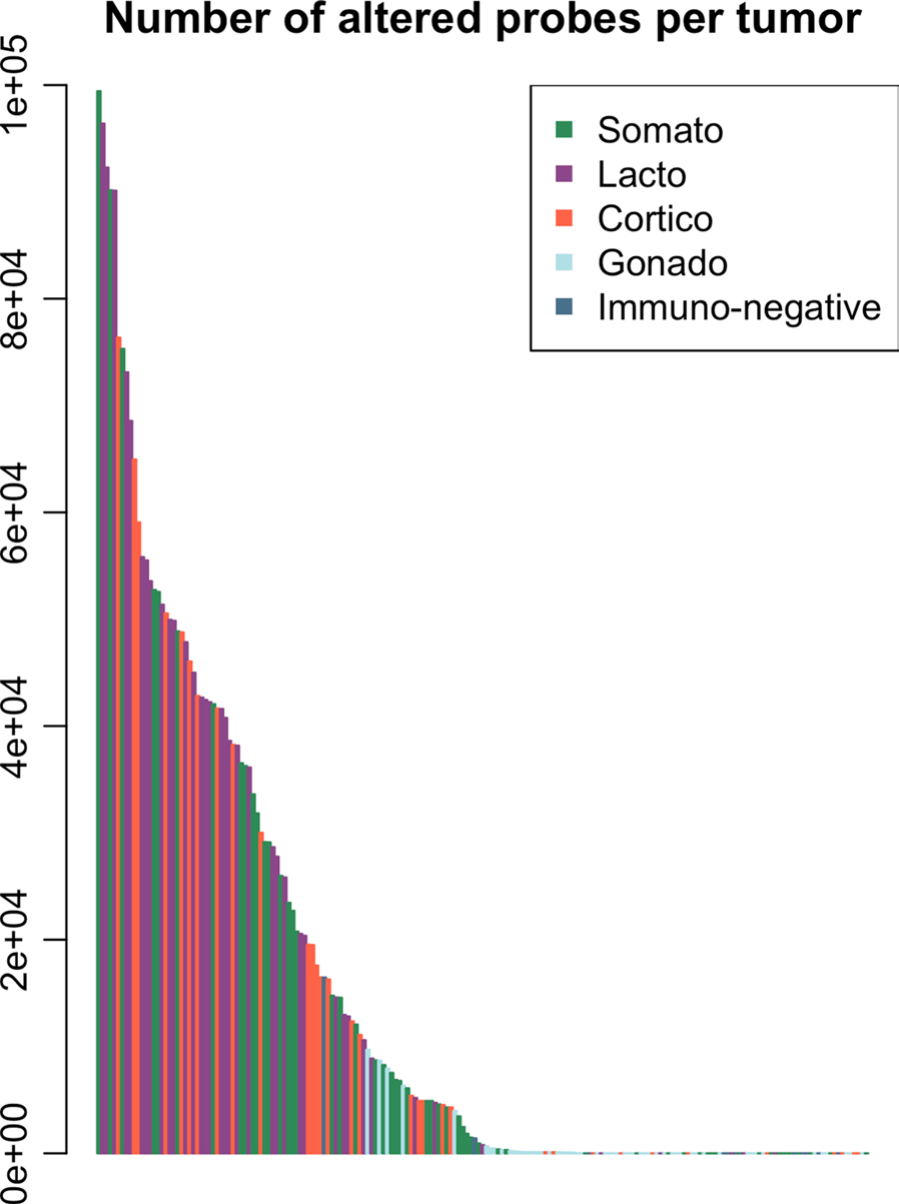
Barplot of the quantities of altered probes per tumor

**Fig. 2:**
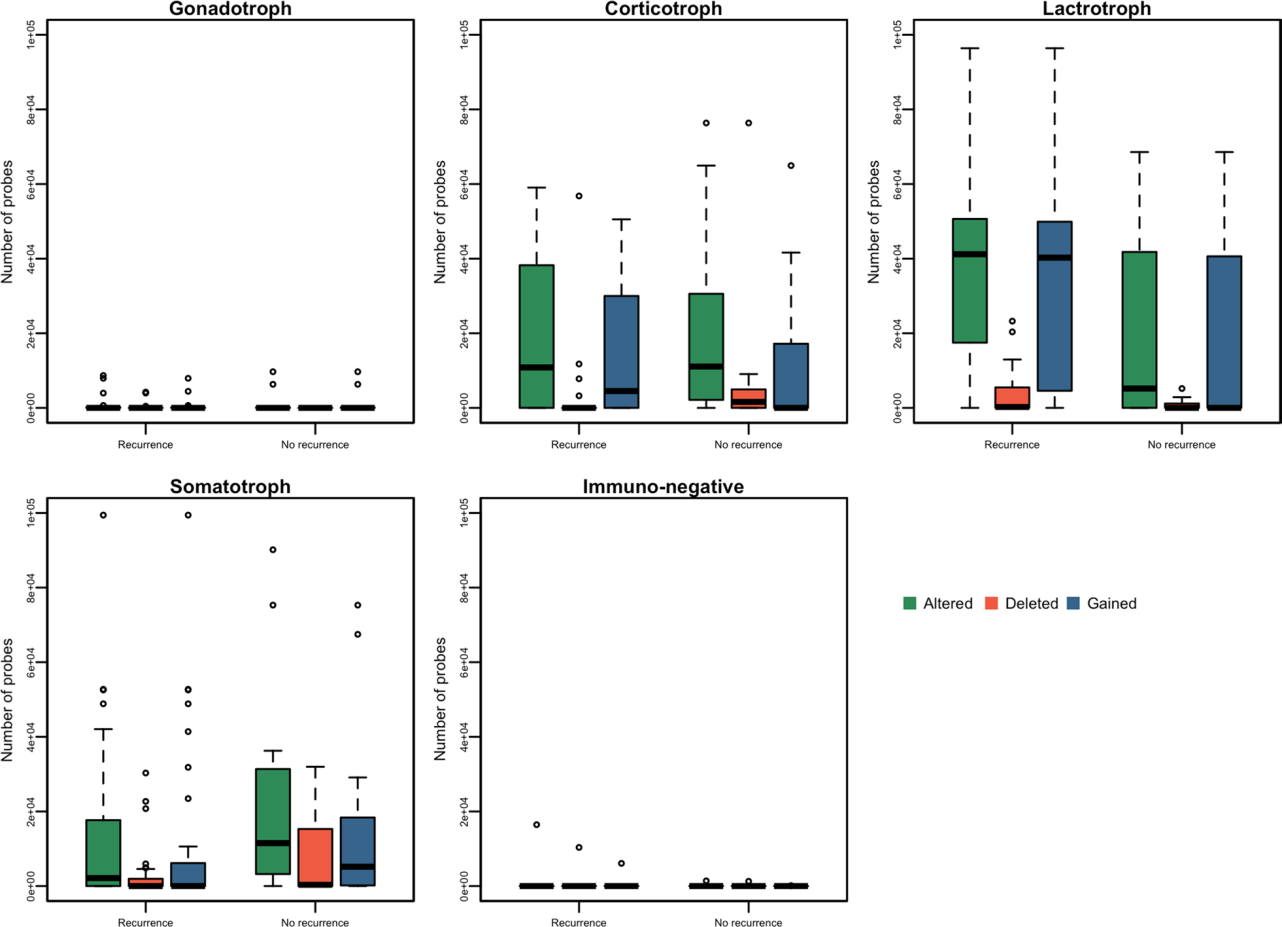
Boxplot of the quantities of altered, deleted and gained probes per tumor type

Genomic instability was dependent on the tumor type (Figs. 1, 2). Median (min–max) percentage of altered probes per tumor (total = 99,659 CGH probes) was 0% (0–9.7) in gonadotroph and 0% (0–16.5) in immunonegative, compared to 4.8% (0–99.8) in somatotroph, 11.1% (0–76.6) in corticotroph and 38.3% (0–96.7) in lactotroph tumors. Gains were globally more frequent than deletions: 0% (0–9.7) versus 0% (0–4.3) in gonadotroph, 0% (0–6.1) versus 0% (0–10.4) in immunonegative, 0.4% (0–99.8) versus 0% (0–32.1) in somatotroph, 2.3% (0–65.2) versus 0% (0–76.6) in corticotroph and 36.2% (0–96.7) versus 0% (0- 23.3) in lactotroph tumors (Fig. 2). LOH were less frequent and detected in 13/56 gonadotroph, 2/11 immunonegative, 17/56 somatotroph, 9/33 corticotroph and 15/39 lactotroph tumors (median 0% for all types). Large alterations of entire chromosomes or chromosome arms were frequent. Entire chromosomes 9, 5, 7, 12, 19, 20 were gained in 41 (21%), 38 (19%), 38, 37 (19%), 37 and 37 patients respectively, while short arms of chromosomes 7, 19, and 9 were gained in 43 (22%), 42 (22%) and 42 (22%) patients respectively. Common deletions were rarely found and whole chromosomes 18, 11 and 13 were deleted in 10 (4%), 9 (3%) and 8 (3%) patients respectively.

In gonadotroph tumors, whole chromosome 7 was gained only in 3/56 patients and no large recurrent alterations were detected in immunonegative tumors. In somatotroph tumors, the large alteration most frequently found concerned chromosome 9, with 14/56 patients showing a whole chromosome gain and 1/56 patients presenting a gain of the entire short arm. In corticotroph tumors, the most frequent large alteration concerned chromosome 12 (gain of the entire chromosome in 11/33 (33%) patients). Regarding lactotroph tumors, gain of whole chromosomes 9, 12, 7 and 19 were found in 21/39, 19/39, 19/39 and 18/39 patients respectively, whereas a gain of their short arms were found in another 0/39, 1/39, 3/39 and 3/39 tumors. Deletion of chromosome 18 was observed in 4/39 lactotroph tumors.

The quantity of altered probe per tumor was extremely variable and no evident threshold could be identified to classify tumors (Fig. 1). Clinical and pathological characteristics of tumors showing alterations, defined as tumors with at least one CNV, compared to tumors lacking CNVs are presented for each tumor type in Table 2.

**Table 2:** Comparison of altered versus non-altered tumors

	Alterations	No alterations
Gonadotroph	24	32
Age	54.19 (14.1)	54 (14.1)
Sex		
F	10 (41.7%)	11 (34.4%)
M	14 (58.3%)	21 (65.6%)
Recurrence		
Yes	15 (62.5%)	18 (56.3%)
No	9 (37.5%)	14 (43.7%)
Grade		
1a	7 (29.2%)	11 (34.4%)
1b	1 (4.2%)	1 (3.1%)
2a	12 (50.0%)	16 (50%)
2b	4 (16.6%)	4 (12.5%)
Size		
Microadenomas	0 (0%)	0 (0%)
Macroadenomas	24 (100%)	31 (96.9%)
Giant Adenomas	0 (0%)	1 (3.1%)
Secretion		
Silent	24 (100%)	32 (100%)
Functioning	0 (0%)	0 (0%)
Immunonegative	2	9
Age	52.49 (7.5)	58.36 (11.6)
Sex		
F	1 (50%)	3 (33.3%)
M	1 (50%)	6 (66.7%)
Recurrence		
Yes	1 (50%)	4 (44.4%)
No	1 (50%)	5 (55.6%)
Grade		
1a	0 (0%)	2 (22.2%)
1b	0 (0%)	1 (11.1%)
2a	2 (100%)	6 (66.7%)
2b	0 (0%)	0 (0%)
Size		
Microadenomas	0 (0%)	0 (0%)
Macroadenomas	2 (100%)	8 (88.9%)
Giant Adenomas	0 (0%)	1 (11.1%)
Secretion		
Silent	2 (100%)	9 (100%)
Functioning	0 (0%)	0 (0%)
Somatotroph	37	19
Age	44.36 (12.9)	41.84 (10.8)
Sex		
F	18 (48.7%)	12 (63.2%)
M	19 (51.3%)	7 (36.8%)
Recurrence		
Yes	23 (62.2%)	17 (89.5%)
No	14 (37.8%)	2 (10.5%)
Grade		
1a	12 (32.4%)	4 (21.1%)
1b	2 (5.4%)	0 (0%)
2a	16 (43.3%)	10 (52.6%)
2b	7 (18.9%)	5 (26.3%)
Size		
Microadenomas	3 (8.1%)	1 (5.6%)
Macroadenomas	33 (89.2%)	17 (94.4%)
Giant Adenomas	1 (2.7%)	0 (0%)
GNAS		
WT	25 (67.6%)	15 (78.9%)
Mutation	10 (27.0%)	3 (15.8%)
Not available	2 (5.4%)	1 (5.3%)
Secretion		
Silent	0 (0%)	4 (21.1%)
Functioning	37 (100%)	15 (78.9%)
Lactrotroph	34	5
Age	42.06 (14.7)	40.1 (6.9)
Sex		
F	11 (32.4%)	2 (40.0%)
M	23 (67.6%)	3 (60.0%)
Recurrence		
Yes	27 (79.4%)	1 (20.0%)
No	7 (20.6%)	4 (80.0%)
Grade		
1a	9 (26.5%)	1 (20.0%)
1b	2 (5.9%)	1 (20.0%)
2a	12 (35.3%)	1 (20.0%)
2b	11 (32.3%)	2 (40.0%)
Size		
Microadenomas	1 (2.9%)	1 (20.0%)
Macroadenomas	28 (82.4%)	4 (80.0%)
Giant Adenomas	5 (14.7%)	0 (0.0%)
Secretion		
Silent	2 (5.9%)	0 (0%)
Functioning	32 (94.1%)	5 (100%)
Corticotroph	24	9
Age	45.99 (12.0)	39.37 (18.7)
Sex		
F	11 (45.8%)	5 (55.6%)
M	13 (54.2%)	4 (44.4%)
Recurrence		
Yes	13 (54.2%)	5 (55.6%)
No	11 (45.8%)	4 (44.4%)
Grade		
1a	12 (50.0%)	4 (44.5%)
1b	1 (4.2%)	0 (0.0%)
2a	11 (45.8%)	3 (33.3%)
2b	0 (0%)	2 (22.2%)
Size		
Microadenomas	5 (20.8%)	2 (22.2%)
Macroadenomas	19 (79.2%)	7 (77.8%)
Giant Adenomas	0 (0.0%)	0 (0.0%)
Secretion		
Silent	5 (20.8%)	3 (33.3%)
Functioning	19 (79.2%)	6 (66.7%)
USP8		
WT	17 (70.8%)	5 (55.6%)
Mutation	4 (16.7%)	1 (11.1%)
Not available	3 (12.5%)	3 (33.3%)

We found that the quantity of altered probes in somatotroph tumors was not associated with *GNAS* mutation and alterations were found in both *GNAS*mut and *GNAS*wt tumors. The median (min–max) of altered probes was 6% (0–15) in *GNAS*mut compared to 5% (0–100) in *GNAS*wt (p-value = 0.57). However, the quantity of altered probes associated with secretion. All 4 silent tumors had no altered probe compared to 6% (0–100) of altered probes in functioning somatotroph tumors (p-value = 0.02).

In lactotroph tumors, the quantity of altered probes did not associate with secretion (median (min–max) of altered probes were 21% (1–42) in silent tumors versus 38% (0–97) in functioning tumors, p-value = 0.5).

For corticotroph tumors, most of the identified alterations concerned macroadenomas, while microadenomas appeared less altered (median (min–max) of altered probes were 20% (0–77) versus 0.1% (0–17)), but this was not statistically significant (p-value = 0.15). The quantity of altered probes was not different between *USP8*mut and *USP8*wt (11% (0–59) versus 17% (0–77); p-value = 0.75), silent and functioning (10% (0–46) versus 11% (0–77); p value = 0.69) or invasive and non-invasive corticotroph tumors (17% (0–77) versus 5% (0–49); p-value = 0.46).

### Non-supervised analysis

**Fig. 3:**
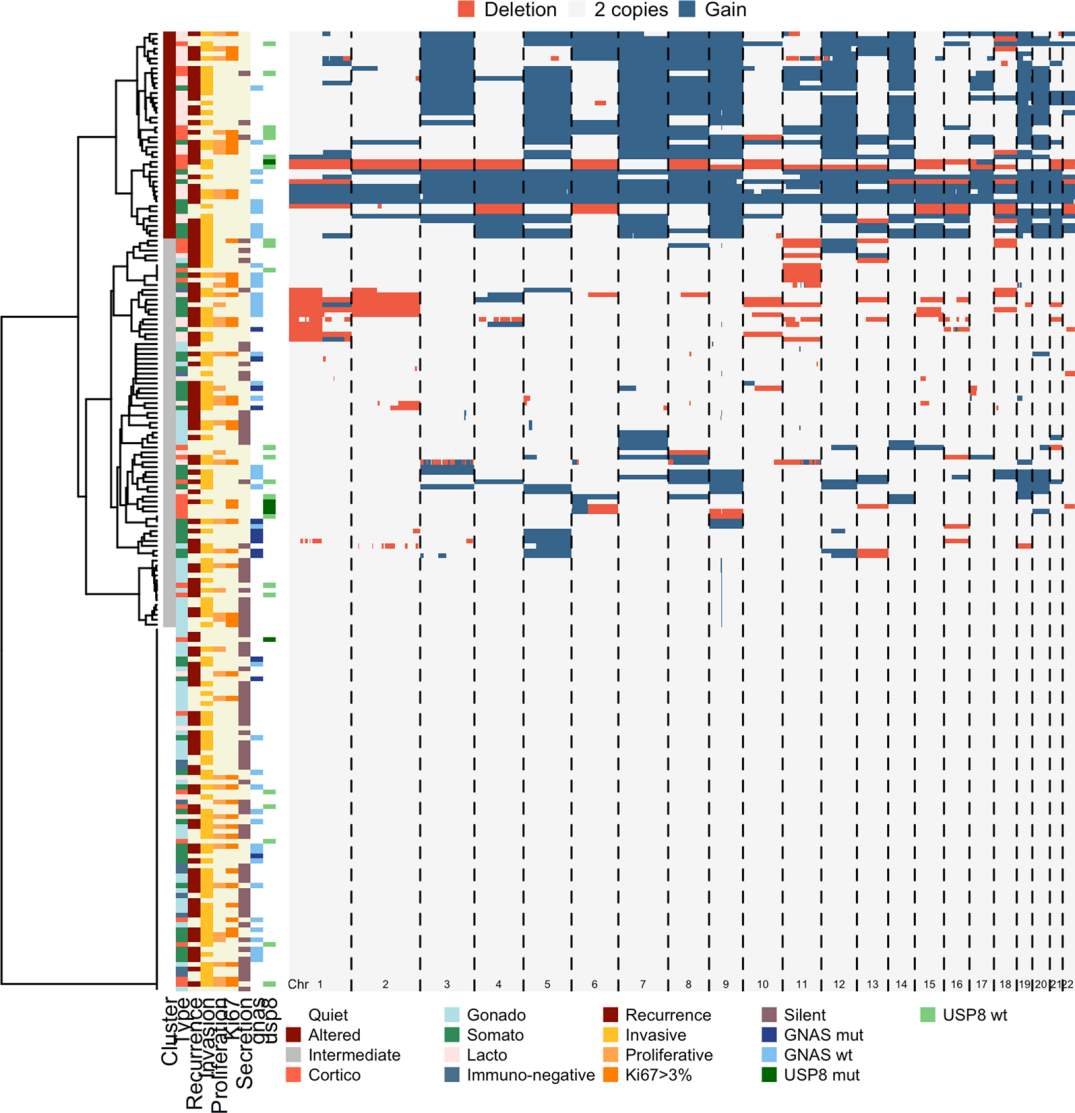
Heatmap of the non-hierarchical clustering in the whole cohort

Clustering analysis performed on the whole cohort identified 3 major clusters based on the number of altered probes (Fig. 3). 74 tumors were included in the ‘quiet’ cluster (no alteration), and 42 tumors were in the most altered cluster (med (min–max) of altered probes = 49% (26–99.8%)). The last 79 tumors were in the intermediate cluster (5% of altered probes (0–37%)). As presented in Fig. 3, while clusters were significantly associated with tumor type (p-value < 0.001), they were not associated with other pathological (grade, invasion, proliferation) or clinical criteria (size, age, sex), confirming that cell lineage was the strongest factor influencing genomic instability. Indeed, no gonadotroph or immuno-negative PitNETs were found in the most altered cluster whereas only 5 lactotroph tumors were in the ‘quiet’ cluster.

### Prognostic analysis

While the quantities of altered, gained, deleted or copy neutral LOH probes were not associated with prognosis in univariate analysis or in multivariate analysis in the whole cohort, the pathological classification (p-value LRT < 0.001) and age at surgery (p-value LRT < 0.001) were associated with prognosis. Hence, grades 2a and 2b were associated with higher risk of recurrence compared to grade 1a in multivariate analysis adjusted for age, sex, and tumor type (OR = 4.3 IC95% [2.1; 9.3] and OR = 8.7 [2.9–30.5] respectively), as well as a younger age at surgery (OR for 10 years older = 0.6 IC95% [0.5;0.8]). The analysis did not reveal an association of specific CNV with tumor recurrence when tested on the whole cohort.

In lactotroph tumors, the quantities of altered (gained and deleted) probes, and more specifically gained probes, were associated with recurrence in univariate analysis (p-value LRT = 0.004 and 0.02 respectively) and multivariate analysis (p-value LRT = 0.003 and 0.02 respectively). In multivariate analysis, the risk of recurrence was multiplied by 1.3 for doubling of altered probes (OR = 1.3 IC95% [1.1;1.6]) and 1.2 for doubling of gained probes (OR = 1.2 IC95% [1.0;1.3]). Similar results were obtained when considering altered probes as a combination of gained, deleted and copy neutral LOH. We also found that the quantities of deleted probes and copy neutral LOH were not individually associated with tumor recurrence. No specific CNV was found to be significantly associated with recurrence after regression on each probe and correction for multiple testing. The number of genes included in the CGHarray alterations in lactotroph tumors was 18,577. The numbers of recurrent and non-recurrent tumors related to the alteration for each gene are listed in Supplemental Table 1. Logistic regression on genes included in the alterations found 2189 genes significantly associated with prognosis after p-value adjustment using univariate analysis, and 1329 using multivariate analysis. The genes and their p-values are listed in Supplemental Table 2. These genes were included in CNV which concerned chromosomes 1–16.

In corticotroph tumors, the quantity of deleted probes tended to be associated with fewer recurrences, though was not statistically significant (OR = 0.9 IC95% [0.8;1.0], p-value LRT = 0.08 in univariate analysis, OR = 0.9 IC95% [0.8;1.0], p-value LRT = 0.07 in multivariate analysis), while *USP8* mutations were not associated with prognosis (p-value LRT = 0.82).

In somatotroph tumors the quantity of altered probes, as well as *GNAS* mutations, were not associated with tumor recurrence. However, while considering tumors with *GNAS* mutation or gain of the *GNAS* region, tumors with none of these alterations were significantly more likely to show recurrence (p-value of univariate LRT = 0.02; OR = 4.2 IC95% [1.2;17.6]). Note, that this association was not found in multivariate analysis (p-value LRT = 0.11).

In gonadotroph and immunonegative tumors, quantity of deleted probes was limited and not associated with tumor recurrence.

**Fig. 4:**
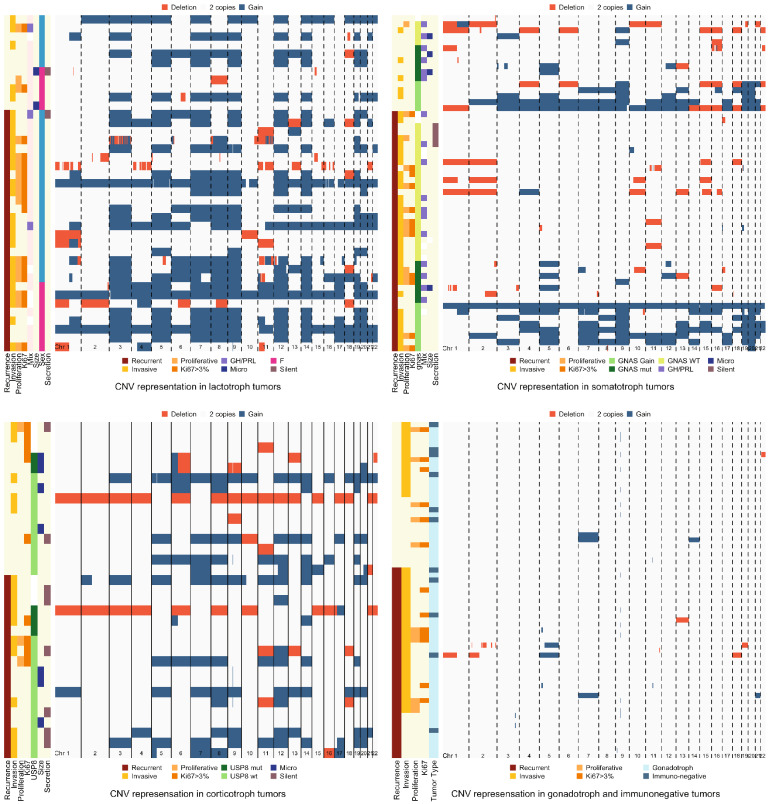
Heatmap representation of the CNV in each tumor type

We did not find specific CNV associated with recurrence after regression on each probe and correction for multiple testing in somatotroph, corticotroph and gonadotroph tumors (Fig. 4).

### CGH array and transcriptomic analysis in lactotroph tumors

To extend our work, we performed a transcriptomic analysis of 32 lactotroph tumors (25 recurrent, 7 non-recurrent, 16 of whom had CGHarray analysis). The top 100 genes associated with recurrent phenotype were ranked according to signal-to-noise ratio (Supplemental Figure 2). No gene sets appeared significantly associated with recurrent phenotype using GSEA analysis based on Hallmark gene sets. Among the 2189 and 1329 genes, which were found altered in lactotroph tumors by CGHarray and associated with prognosis by univariate and multivariate analysis, 40 and 29 were significantly differentially expressed between recurrent and non-recurrent tumors in our transcriptomic analysis respectively (Table 3). These genes localized mostly on chromosomes 1 and 11.

**Table 3:** List of genes associated with recurrence in lactotroph tumors selected using CGH array (univariate and multivariate analysis) and transcriptomic analysis

Gene	Chromosome	aCGH univariate logistic regressions		aCGH multivariate logistic regressions^b^		Transcriptomic analysis	
		LRT raw p-values	LRT adjusted p-values^a^	LRT raw p-values	LRT adjusted p-values^a^	Fold-Change	T-test p-values
***ACTL8***	**1**	**0.003**	**0.041**	**0.003**	**0.046**	**10.955**	**0.004**
*ALDH3B1*	11	0.005	0.041	0.012	0.099	2.194	0.033
***AMBRA1***	**11**	**0.002**	**0.041**	**0.002**	**0.046**	**3.642**	**0.033**
***ATPAF1***	**1**	**0.005**	**0.041**	**0.003**	**0.046**	− **2.104**	**0.016**
***CEL***	**9**	**0.001**	**0.041**	**0.003**	**0.046**	**2.392**	**0.004**
***CLCNKA***	**1**	**0.003**	**0.041**	**0.003**	**0.046**	**9.043**	**0.012**
*DLG2*	11	0.005	0.041	0.012	0.099	− 2.146	0.027
***DOCK7***	**1**	**0.005**	**0.041**	**0.003**	**0.046**	**2.100**	**0.000**
*DPAGT1*	11	0.005	0.041	0.012	0.099	6.217	0.001
***ELAVL4***	**1**	**0.005**	**0.041**	**0.003**	**0.046**	**2.786**	**0.039**
***FPGT*-*TNNI3K***	**1**	**0.003**	**0.041**	**0.003**	**0.046**	**2.064**	**0.036**
*FRMD8*	11	0.005	0.041	0.012	0.099	25.132	0.007
***GSTM4***	**1**	**0.003**	**0.041**	**0.003**	**0.046**	**19.111**	**0.019**
***HECTD3***	**1**	**0.005**	**0.041**	**0.003**	**0.046**	**2.465**	**0.002**
***HMGCL***	**1**	**0.001**	**0.041**	**0.003**	**0.046**	− **2.135**	**0.017**
***HSD17B12***	**11**	**0.002**	**0.041**	**0.002**	**0.046**	− **2.021**	**0.016**
***HSPB7***	**1**	**0.003**	**0.041**	**0.003**	**0.046**	**10.999**	**0.012**
***HTATIP2***	**11**	**0.002**	**0.041**	**0.002**	**0.046**	− **2.014**	**0.008**
*KCNJ5*	11	0.005	0.041	0.012	0.099	− 2.514	0.002
***LAPTM5***	**1**	**0.003**	**0.041**	**0.003**	**0.046**	− **2.272**	**0.034**
***LPL***	**8**	**0.000**	**0.041**	**0.001**	**0.046**	**2.578**	**0.003**
***MAST2***	**1**	**0.005**	**0.041**	**0.003**	**0.046**	**2.094**	**0.023**
***MDK***	**11**	**0.002**	**0.041**	**0.002**	**0.046**	− **2.180**	**0.041**
***NRIP3***	**11**	**0.002**	**0.041**	**0.002**	**0.046**	**2.076**	**0.020**
***PAMR1***	**11**	**0.002**	**0.041**	**0.002**	**0.046**	− **2.563**	**0.048**
*PGM2L1*	11	0.005	0.041	0.012	0.099	2.222	0.013
*PHOX2A*	11	0.005	0.041	0.012	0.099	52.058	0.007
***PIFO***	**1**	**0.003**	**0.041**	**0.003**	**0.046**	**2.751**	**0.001**
***PLA2G2F***	**1**	**0.003**	**0.041**	**0.003**	**0.046**	**3.403**	**0.047**
***POMGNT1***	**1**	**0.005**	**0.041**	**0.003**	**0.046**	**15.479**	**0.047**
***PRKAA2***	**1**	**0.005**	**0.041**	**0.003**	**0.046**	**2.568**	**0.002**
***RHCE***	**1**	**0.003**	**0.041**	**0.003**	**0.046**	− **2.184**	**0.017**
*RPUSD4*	11	0.005	0.041	0.012	0.099	2.011	0.001
***RUNX3***	**1**	**0.003**	**0.041**	**0.003**	**0.046**	− **2.438**	**0.003**
*SCGB1D2*	11	0.005	0.041	0.012	0.099	2.155	0.005
***SOX6***	**11**	**0.001**	**0.041**	**0.001**	**0.046**	**37.554**	**0.000**
*SRSF8*	11	0.005	0.041	0.012	0.099	− 2.223	0.010
***ST3GAL3***	**1**	**0.005**	**0.041**	**0.003**	**0.046**	**11.519**	**0.000**
***TARDBP***	**1**	**0.003**	**0.041**	**0.003**	**0.046**	**2.117**	**0.001**
*TMEM216*	11	0.005	0.041	0.012	0.099	− 2.006	0.009

Bold character correspond to significant adjusted p-values of aCGH multivariate logistic regressions

*LRT* likelihood ratio test

^a^Benjamini-Hochberg

^a^Adjusted for age at diagnosis, sex, histologic grade

## Discussion

Here, we report the first study of a large multicentric cohort of PitNETs patients with standardized clinical follow-up, clear definition of recurrence and available pathology data, in which we analyzed the impact of chromosome instability on tumor prognosis. As shown previously [4, 16], our CGHarray results confirmed the large number of CNV that can be detected in PitNETs. We report that the amount of genome alteration is associated with tumor types but not with the prognosis in the whole cohort. However, our data also support that the number of genomic alterations found in lactotroph tumors are associated with a poor prognosis, independently of the tumor’s invasive and proliferative status.

We observed a wide range of proportions of altered genome, varying from 0 to almost 100% of the whole genome. While gains were more frequently observed compared to deletions, copy neutral LOH were rare compared to CNV. This result is rather surprising for this type of frequently indolent tumor, in view of large numbers of genomic alterations being generally a key feature of more aggressive tumors with metastatic spread [11]. Unlike other studies [4, 9, 16, 26], we did not find a clear threshold to categorize PitNETs as “altered” or “quiet” in our entire cohort, nor in each tumor type, as the quantity of altered genome per tumor was continuous.

Analysis of prognosis for the whole cohort did not show any association between the quantity of alterations or specific CNV, and 5 years’ recurrence status. As previously reported, histological classification, which associates invasiveness and proliferation criteria (Ki67 index, p53 expression and mitotic index), was associated with prognosis, as well as age at surgery [22, 32].

Interestingly, using univariate analysis and multivariate analysis adjusted for age, sex, and histological classification, we found that the quantity of alterations was an independent risk factor for recurrence for lactotroph tumors. However, the exact consequences of these alterations that underlie recurrence remain unclear. Alterations of chromosomes 1p, 11 and 17 were exclusively found in recurrent tumors however, we did not find CNV statistically associated with recurrence after adjustment of p-values. We cannot exclude the possibility that the combination of a series of specific CNVs may lead to an increased risk of recurrence, while a lack of power regarding our study should not be excluded. The number of genes included in the alterations being high, we failed to identify specific and relevant target genes using CGHarray analysis. It is important to emphasize that the consequences of CNV on gene expression are difficult to predict, especially in the case of gains, which can localize anywhere on the genome and impact the functionality of long range enhancers and silencers. We evaluated the impact of CNV on gene expression thanks to transcriptomic analysis of 32 lactrotroph tumors. Through this approach, we found 29 genes, among the 1329 identified with CGHarray using multivariate analysis, that were significantly differentially expressed between recurrent tumors versus non-recurrent tumors. However, further analysis of these 29 candidates, did not identify an over-represented pathway.

Various genes have been suspected to be associated with PitNETs aggressiveness. While *TP53* mutations have been described in pituitary carcinomas [29], no deletions including *TP53* were found in lactotroph tumors in our cohort. Mutations of the protoconcogene *HRAS* have been reported in metastasis [20] and alterations including *HRAS* (chromosome 11) were found in 8 recurrent lactotroph tumors with various mechanism (4 gains, 3 deletions, 1 copy neutral LOH), while expression was not different in recurrent tumors. Lastly, reduced expression of D2R is suspected to be associated with dopamine agonist resistance in prolactinomas [37]. Here, we found the *DRD2* gene deleted and in copy neutral LOH in 5 and 2 recurrent tumors respectively. However, *DRD2* expression was not different in our transcriptomic analysis. The consequences of these CNV are thus difficult to evaluate.

Our results underline that the quantity of alterations is associated with PitNET type. Whereas lactotroph tumors were the most altered tumors, gonadotroph and non-immunoreactive tumors only present a small number of short CNVs.

Bi et al. [4], Salomon et al. [24] and Neou et al. [16] also described an association between functional characteristics and quantity of alterations whereas Song et al. did not [26]. In these studies, non-functional tumors included not only gonadotroph and immunonegative tumors, but also silent corticotroph and, occasionally, thyreotroph, somatotroph and lactotroph tumors. The mechanisms of tumorigenesis of the gonadotroph and immunonegative tumors remain unclear as they present a few short CNV and no mutations in sequencing studies [17].

One may also question why some corticotroph and somatotroph tumors were found to present no alterations. Similar results have been previously reported by others [4, 7, 9, 16, 24], while the association with secretory phenotype [4, 16], and the role of PTTG1 [27] or hypomethylation [16] have also been suggested.

In our cohort, 8/33 corticotroph tumors were clinically silent and the aCGH profile of these tumors was not distinguishable from those of the clinically functional corticotroph tumors. 9/33 corticotroph tumors showed no alteration, whereas 9/33 were in the most altered cluster. We found no association between alteration numbers/clusters and tumor invasion, tumor recurrence or *USP8* mutations. We did not find the quantity of alterations in *USP8*wt tumors to be associated with tumor invasion, unlike the findings of Tatsi et al. [30]. However, the small number of invasive *USP8*wt corticotroph tumors present in our cohort (n = 9) and in the Tatsi et al. cohort (n = 2), means that our results should be taken with caution [30]. In accordance with Tatsi et al, larger tumors seemed to be more altered though this did not reach statistical significance in our study.

Contrary to Hage et al. and Valimaki et al., we did not find a significant association between *GNAS* mutations (found in 13/53 tumors) and the quantity of genomic alterations in somatotroph tumors [9, 33]. We identified 13 *GNAS*wt tumors presenting gains of the 20q region, these tumors further presenting a high quantity of alterations. On the contrary, none of the *GNAS* mutated tumors harbored gains of the 20q region. Duplication of the *GNAS* gene has been proposed as an alternative mechanism in somatotroph tumorigenesis [9]. Neou et al. [16] described that *GNAS* mutated tumors were associated with fewer chromosomal alterations and DNA hypomethylation, whereas hypomethylation was associated with chromosomal alterations in other *POU1F1*/PIT1 lineage tumors. Data regarding the association between *GNAS* mutation and prognosis in the literature are inconsistent [8, 12, 34]. In our study, *GNAS* mutation was not associated with recurrences. However, somatotroph tumors with *GNAS* mutation or gain of the *GNAS* region presented significantly less recurrence than tumors lacking *GNAS* alteration by univariate analysis.

While our conclusion is appealing, some caution is required due to study limitations, such as the detection sensitivity of the CNV which may be affected through the pre-treatment of our CGH data. Hence the Log2(ratio) threshold to define a gain or a deletion is particularly important. A more sensitive threshold allows the detection of small cell populations, but also risks exposure to artifacts and false discoveries. Moreover, lactotroph tumors included in our study are not representative of the usual clinical presentation. Most indolent lactotroph lesions are medically managed and most that are operated are associated with an aggressive behavior. This could partly explain the high quantity of alterations observed in those tumors. In addition, macro-corticotroph tumors are likely overrepresented whereas typical microadenomas may be underrepresented due to the limited material available for analysis.

In conclusion, our study confirms the association between genomic alterations and PitNET type, suggesting that mechanisms associated with pituitary tumorigenesis and behavior are specific for each tumor type. In lactotroph tumors, genomic instability can partly explain tumorigenesis and mechanism of progression, whereas the mechanism of tumorigenesis and recurrence in gonadotroph tumors remains unclear and requires further exploration of other mechanisms, such as the role of the micro-environment, epigenetic mechanisms and cellular heterogeneity.

## Supplementary information

Additional file 1

Additional file 2

Additional file 3

## Data Availability

CGHarray data will be available at the EMBL-EBI Array Express under accession number E-MTAB-9237. Transcriptomic data are available at Gene Expression Omnibus (GEO) under accession number GSE120350.
