# Supplementary material for: Chromosomal instability in the prediction of pituitary neuroendocrine tumors prognosis
Source: Acta Neuropathol Commun. 2020 Nov 10;8:190. doi: 10.1186/s40478-020-01067-5 (PMC7653703; doi:10.1186/s40478-020-01067-5)
Supplement: Supplementary file 1 — Additional file 1 [file 40478_2020_1067_MOESM1_ESM.pdf]

Chromosomal instability in the prediction of pituitary tumor prognosis.

Hélène Lasolle, Mad-Hélénie Elsensohn, Anne Wierinckx, Eudeline Alix, Clément Bonnefille, Alexandre Vasiljevic, Christine Cortet, Bénédicte Decoudier, Nathalie Sturm, Stephan Gaillard, Amandine Ferrière, Pascal Roy, Emmanuel Jouanneau, Philippe Bertolino, Claire Bardel, Damien Sanlaville, Gérald Raverot*.

*Corresponding author:

Gérald Raverot

Fédération d'endocrinologie

Centre de Référence des Maladies Rares Hypophysaires

Groupement Hospitalier Est, Hospices civils de Lyon

gerald.raverot@chu-lyon.fr

Supplementary material

Table S1: Number of recurrent and non-recurrent lactotroph tumors concerned by deletion, gain and copy-neutral LOH for each altered gene.
Table S2: LRT p-values of univariate and multivariate logistic regressions on the genes significantly associated with recurrence in lactotroph tumors.

Figure S1 : FISH analysis in 8 tumors harboring gains of the GNAS locus.

A. Heatmap representation of the CNV in the somatotroph tumors with GNAS locus duplication which were analyzed using FISH.
B. Illustration of the results obtained with FISH analysis

FISH was performed on interphasic nuclei from frozen tumor-tissue appositions. Probes used for FISH studies included 11pter (D11S2071) subtelomere probe (Cytocell, Cambridge, UK) in green and BAC RP1-309F20 encompassing *GNAS* locus in red. Independent analyses of more than 20 cells were carried out for each tumor. For tumor 1 to 5, at least 3 red dots are observed in each nucleus confirming the *GNAS* locus duplication identified by CGHarray. FISH analysis confirmed chromosome 11 gains in tumor 2 and tumor 4, as shown using CGHarray**.** For tumor 6 to 8, FISH analysis confirmed the absence of chromosome 20 and chromosome 11 gains. Tumor 6 and 8 harbored *GNAS* mutation while tumor 7 did not.

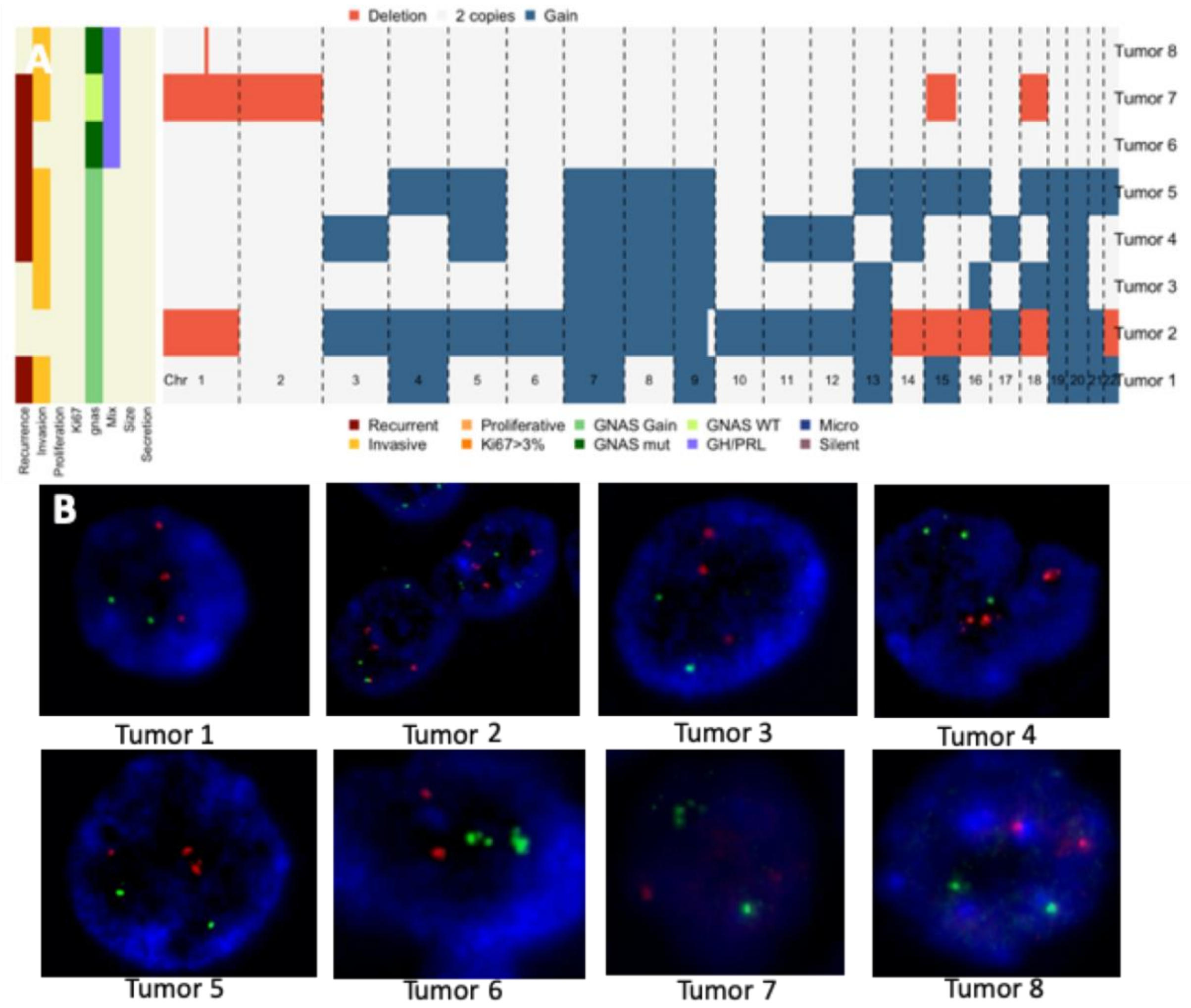

Figure S2: Heatmap representation of the transcriptomic analysis of 32 lactotroph tumors using GSEA with hallmark gene set (100 top genes associated with recurrent tumors in grey and non-recurrent in yellow. Upregulated genes are presented in blue, downregulated genes are presented in red)

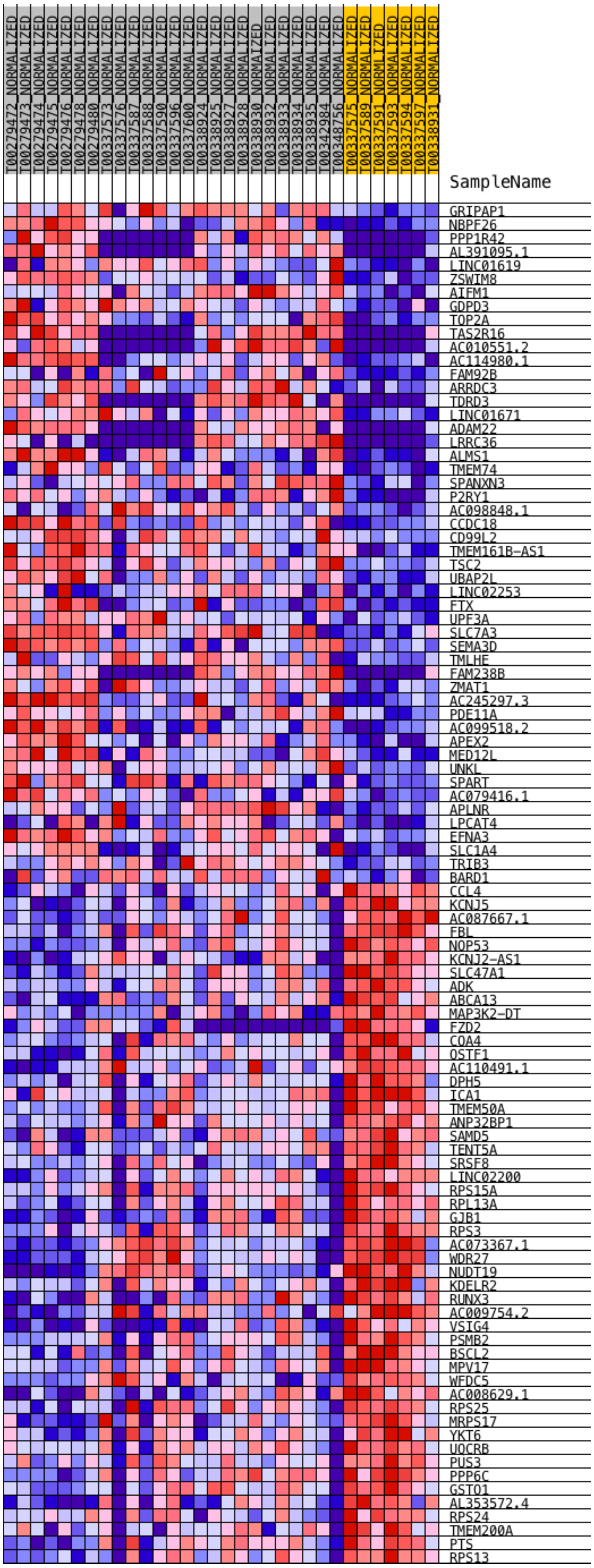

T00279472_NORMALIZED
T00279473_NORMALIZED
T00279474_NORMALIZED
T00279475_NORMALIZED
T00279476_NORMALIZED
T00279478_NORMALIZED
T00279480_NORMALIZED
T00337573_NORMALIZED
T00337576_NORMALIZED
T00337587_NORMALIZED
T00337588_NORMALIZED
T00337590_NORMALIZED
T00337596_NORMALIZED
T00337600_NORMALIZED
T00338924_NORMALIZED
T00338925_NORMALIZED
T00338927_NORMALIZED
T00338928_NORMALIZED
T00338930_NORMAIZED
T00338932_NORMALIZED
T00338933_NORMALIZED
T00338934_NORMALIZED
T00338935_NORMALIZED
T00342984_NORMALIZED
T00348756_NORMALIZED
T00337575_NORMALIZED
T00337589_NORMALIZED
T00337591_NORMLIZED
T00337593_NORMALIZED
T00337594_NORMALIZED
T00337597_NORMALIZED
T00338931_NORMALIZED
SampleName
GRIPAP1
NBPF26
PPP1R42
AL391095.1
LINC01619
ZSWIM8
AIFM1
GDPD3
TOP2A
TAS2R16
AC010551.2
AC114980.1
FAM92B
ARRDC3
TDRD3
LINC01671
ADAM22
LRRC36
ALMS1
TMEM74
SPANXN3
P2RY1
AC098848.1
CCDC18
CD99L2
TMEM161B-AS1
TSC2
UBAP2L
LINC02253
FTX
UPF3A
SLC7A3
SEMA3D
TMLHE
FAM238B
ZMAT1
AC245297.3
PDE11A
AC099518.2
APEX2
MED12L
UNKL
SPART
AC079416.1
APLNR
LPCAT4
EFNA3
SLC1A4
TRIB3
BARD1
CCL4
KCNJ5
AC087667.1
FBL
NOP53
KCNJ2-AS1
SLC47A1
ADK
ABCA13
MAP3K2-DT
FZD2
COA4
OSTF1
AC110491.1
DPH5
ICA1
TMEM50A
ANP32BP1
SAMD5
TENT5A
SRSF8
LINC02200
RPS15A
RPL13A
GJB1
RPS3
AC073367.1
WDR27
NUDT19
KDELR2
RUNX3
AC009754.2
VSIG4
PSMB2
BSCL2
MPV17
WFDC5
AC008629.1
RPS25
MRPS17
YKT6
UQCRB
PUS3
PPP6C
GSTO1
AL353572.4
RPS24
TMEM200A
PTS
RPS13
